# MnO_2_ nanosheets as a carrier and accelerator for improved live-cell biosensing application of CRISPR/Cas12a†

**DOI:** 10.1039/d1sc06383a

**Published:** 2022-03-21

**Authors:** Dong-Xia Wang, Ya-Xin Wang, Jing Wang, Jia-Yi Ma, Bo Liu, An-Na Tang, De-Ming Kong

**Affiliations:** State Key Laboratory of Medicinal Chemical Biology, Tianjin Key Laboratory of Biosensing and Molecular Recognition, Research Centre for Analytical Sciences, College of Chemistry, Nankai University Tianjin 300071 P. R. China kongdem@nankai.edu.cn; School of Pharmacy, Binzhou Medical University Yantai Shandong 264003 PR China

## Abstract

Besides gene-editing, the CRISPR/Cas12a system has also been widely used in *in vitro* biosensing, but its applications in live-cell biosensing are rare. One reason is lacking appropriate carriers to synchronously deliver all components of the CRISPR/Cas12a system into living cells. Herein, we demonstrate that MnO_2_ nanosheets are an excellent carrier of CRISPR/Cas12a due to the two important roles played by them. Through a simple mixing operation, all components of the CRISPR/Cas12a system can be loaded on MnO_2_ nanosheets and thus synchronously delivered into cells. Intracellular glutathione (GSH)-induced decomposition of MnO_2_ nanosheets not only results in the rapid release of the CRISPR/Cas12a system in cells but also provides Mn^2+^ as an accelerator to promote CRISPR/Cas12a-based biosensing of intracellular targets. Due to the merits of highly efficient delivery, rapid intracellular release, and the accelerated signal output reaction, MnO_2_ nanosheets work better than commercial liposome carriers in live-cell biosensing analysis of *survivin* messenger RNA (mRNA), producing much brighter fluorescence images in a shorter time. The use of MnO_2_ nanosheets might provide a good carrier for different CRISPR/Cas systems and achieve the rapid and sensitive live-cell biosensing analysis of different intracellular targets, thus paving a promising way to promote the applications of CRISPR/Cas systems in living cells.

## Abstract 2

Herein, we demonstrate that MnO_2_ nanosheets are an excellent carrier of CRISPR/Cas12a due to the two important roles played by them.

## Graphical abstract

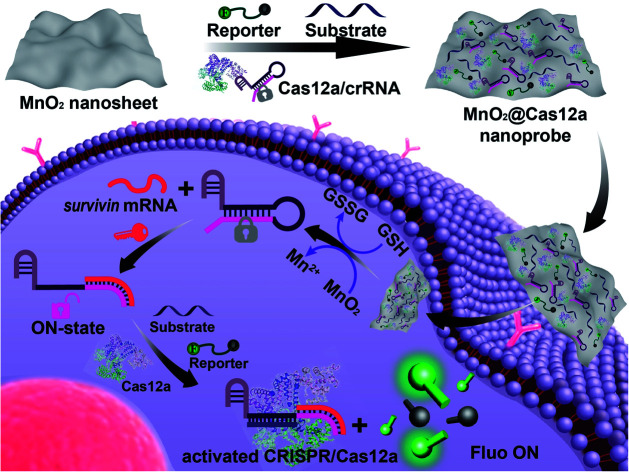

## Introduction

CRISPR (Clustered Regularly Interspaced Short Palindromic Repeats)/Cas (CRISPR-associated) technology has already become a powerful tool for genome manipulation in living cells and organisms.^1–3^ It also provides some exciting opportunities for other applications, *e.g.*, biosensing and bioimaging.^4^ Nearly all single-effector CRISPR/Cas systems, including CRISPR/Cas9, CRISPR/Cas12, CRISPR/Cas13, and CRISPR/Cas14, discovered so far have demonstrated their advantages for developing advanced diagnostic methods.^5–8^ Accompanied by the explosive growth of CRISPR/Cas-based *in vitro* biosensing research, their applications in living cells have become a research hotspot.^9–11^ However, the reported examples are mainly focused on the CRISPR/Cas9 system. Compared to Cas9 which needs a relatively long guide RNA (gRNA), Cas12 needs a much shorter CRISPR RNA (crRNA).^12,13^ More interestingly, besides inherent *cis*-cleavage activity, Cas12 shows an additional *trans*-cleavage activity that can cleave single-stranded DNA (ssDNA) with an arbitrary sequence.^14,15^ These characteristics greatly increase the diversity and flexibility in Cas12-based sensor design.^16–19^ Therefore, compared to Cas9, Cas12 is more often used in *in vitro* biosensing applications recently. However, its live-cell applications are still in their infancy stage.

Currently, the application of CRISPR/Cas12 in living cells is facing two major difficulties: limited activity and poor internalization by living cells. For example, the *cis*-cleavage activity of CRISPR/Cas12a is only one turnover. Its *trans*-cleavage activity was first reported as 1250 turnovers per second and then corrected as ∼17 turnovers per second,^14^ which is not sufficient for the detection of the targets with low expression levels. In biosensing applications, the low activity can be overcome by combining various nucleic acid amplification techniques.^20–22^ However, such a strategy is unsuitable for live-cell applications due to the difficulty of performing the amplification reactions in living cells, greatly delayed signal output, and possible disturbances to the intracellular physiological environment and target expression levels. Therefore, there is an urgent need to develop a simple, enzyme-free, and transfection-free strategy to improve CRISPR/Cas12a-based biosensing performance in living cells. Recently, Ma *et al.* reported that Mn^2+^ could be used as the accelerator of CRISPR/Cas12a *trans*-cleavage activity, giving up to 13-fold sensitivity improvement for CRISPR/Cas12a-based biosensors.^23^ This discovery may provide a simple and feasible way to promote the biosensing performance of CRISPR/Cas12a in living cells.

Since multiple components, including Cas12a protein, crRNA, DNA substrates, and fluorescent DNA probes (reporter), are required by the CRISPR/Cas12a-based sensing system, it is not an easy task to achieve the efficient and synchronous delivery of them into living cells. To the best of our knowledge, only gold nanoparticles and DNA nanoclew (NC)-based carriers were reported for the delivery of Cas12a protein and crRNA (substrate and reporter are not included) for gene-editing.^24,25^ There is still a lack of applicable carriers for CRISPR/Cas12a-based live-cell analysis.

As ultrathin two-dimensional nanomaterials, MnO_2_ nanosheets have attracted remarkable attention in biological applications due to their large specific surface area, excellent biocompatibility, and low toxicity.^26^ It has been demonstrated that MnO_2_ nanosheets can serve as excellent carriers for the delivery of nucleic acids, drugs and nanoprobes into living cells.^26,27^ More interestingly, after being swallowed into cells, MnO_2_ nanosheets can be decomposed by intracellular reducing substances (*e.g.*, overexpressed glutathione (GSH) in cancer cells), resulting in the rapid and highly efficient release of cargoes. Due to these attractive characteristics, MnO_2_ nanosheets hold great promise as carriers of CRISPR/Cas systems for living cell applications.

**Scheme 1:**
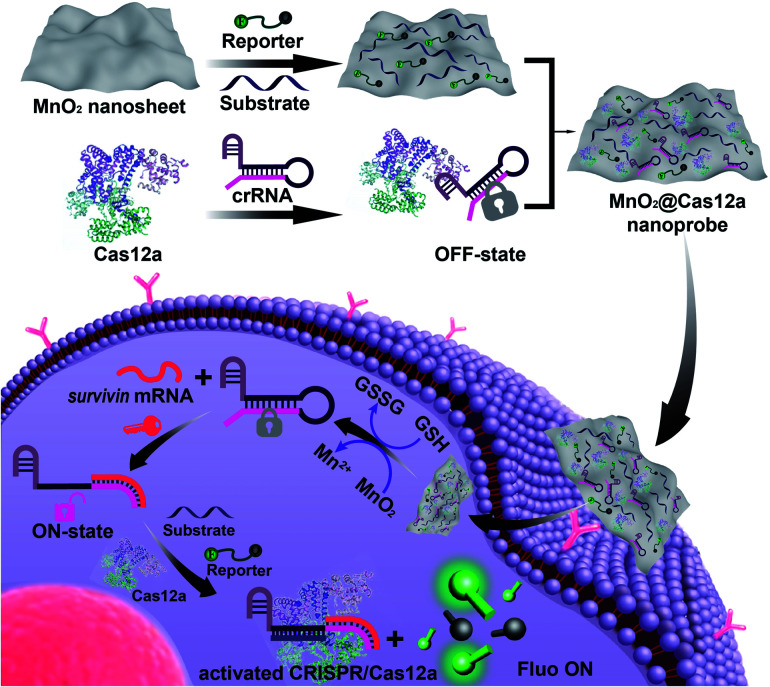
Preparation of the MnO_2_@Cas12a nanoprobe for live-cell analysis of *survivin* mRNA. *Via* physisorption, π-stacking, and electrostatic interactions, substrate/reporter/crRNA/Cas12a can be efficiently assembled on MnO_2_ nanosheets through a simple mixing operation. The MnO_2_@Cas12a can be efficiently internalized by cells to perform *survivin* mRNA-specific biosensing analysis. The processes are as follows: endocytosis and endosome escape, decomposition of MnO_2_ nanosheets by GSH to release the CRISPR/Cas12a system and produce Mn^2+^, target mRNA-mediated activation of CRISPR/Cas12a, and Mn^2+^-accelerated target mRNA analysis.

Herein, we demonstrated that MnO_2_ nanosheets are indeed an excellent carrier of CRISPR/Cas12a. Through a simple mixing operation, all the components of the CRISPR/Cas12a-based sensing system are easily assembled on the surface of MnO_2_ nanosheets to form a MnO_2_@Cas12a nanoprobe that could be efficiently internalized by living cells (Scheme 1). Since MnO_2_ can be rapidly decomposed by intracellular GSH to produce a large amount of Mn^2+^, the MnO_2_ nanosheets play two important roles: delivering the CRISPR/Cas12a system into cells and providing Mn^2+^ to accelerate target-specific sensing performance. The as-designed MnO_2_@Cas12a nanoprobe was demonstrated to work well for the biosensing analysis of target messenger RNA (mRNA) in living cells, with better performance than commercial carriers.

## Results and discussion

### Preparation and characterization of the MnO_2_@Cas12a nanoprobe

**Fig. 1:**
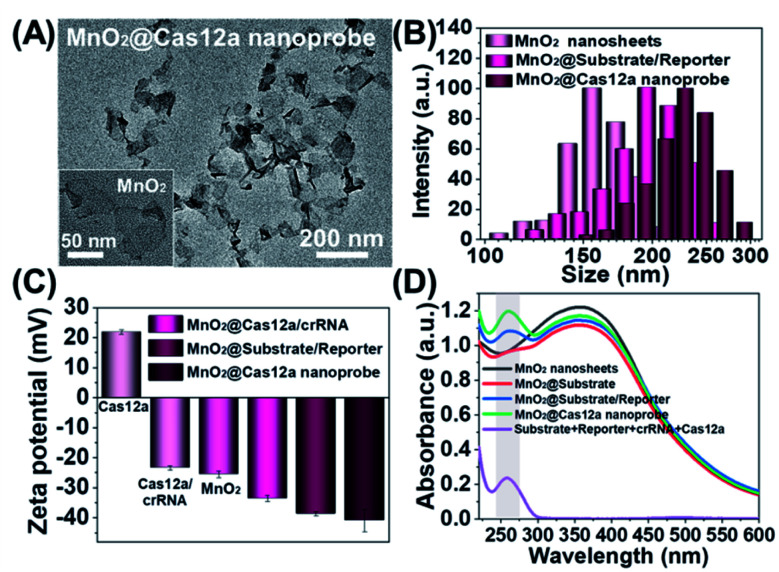
Characterization of the MnO_2_@Cas12a nanoprobe. (A) Transmission electron microscopy (TEM) images of the MnO_2_@Cas12a nanoprobe and MnO_2_ nanosheets (inset). (B) Hydrodynamic size distribution, (C) zeta potential, and (D) UV-Vis absorption spectra of the MnO_2_ nanosheets, MnO_2_@Cas12a nanoprobe and some intermediates.

Our nanoprobe was facilely prepared by directly mixing MnO_2_ nanosheets with the components of the CRISPR/Cas12a-based sensing system including one RNA strand (crRNA, Table S1†), two DNA strands (substrate and reporter, Table S1†), and Cas12a protein. MnO_2_ nanosheets were synthesized through oxidation of MnCl_2_·4H_2_O by H_2_O_2_ in the presence of tetramethylammonium hydroxide (TMA·OH).^28^ The prepared MnO_2_ nanosheets exhibited a sheet-like structure with irregular folds (Fig. 1A, inset), whose average size and zeta potential were approximately 156 ± 36.89 nm (Fig. 1B) and −25.55 ± 2.56 mV (Fig. 1C), respectively. A polydispersity index (PDI) of 0.327 was obtained, thus proving the good dispersity of the MnO_2_ nanosheets. *Via* physisorption and π-stacking interaction, DNA and RNA, including substrate and reporter, could be easily assembled on the surface of MnO_2_ nanosheets, which was reflected by the increase of hydrodynamic size, the decrease of zeta potential, and the coexistence of the characteristic absorption of MnO_2_ centered at 380 nm and DNA (and RNA) at 260 nm (Fig. 1D). Due to the opposite charges of Cas12a protein (21.85 ± 0.78 mV) and the assembly formed by MnO_2_ and DNA/RNA, *via* electrostatic interaction, the Cas12a/crRNA complex could be further assembled to form the nanoprobe of MnO_2_@substrate/reporter/crRNA/Cas12a (abbreviated as the MnO_2_@Cas12a nanoprobe). By comparing the UV-Vis absorption spectra of the substrate/reporter/crRNA/Cas12a mixture before and after assembly with MnO_2_ nanosheets, it could be calculated that about 80% of the CRISPR/Cas12 system was effectively assembled on MnO_2_ nanosheets (Fig. S1†). The prepared MnO_2@Cas12a_ nanoprobe also showed a sheet-like structure (Fig. 1A), giving a zeta potential of −40.95 ± 3.78 mV and hydrodynamic diameter of 231 ± 41.47 nm. Such a high electronegativity assures the high stability of the nanoprobe in biological environments. The homogenous and nanoscale size makes the highly efficient internalization of the nanoprobe by living cells possible.

### Feasibility analysis of MnO_2_/Cas12a for target mRNA-sensing

**Fig. 2:**
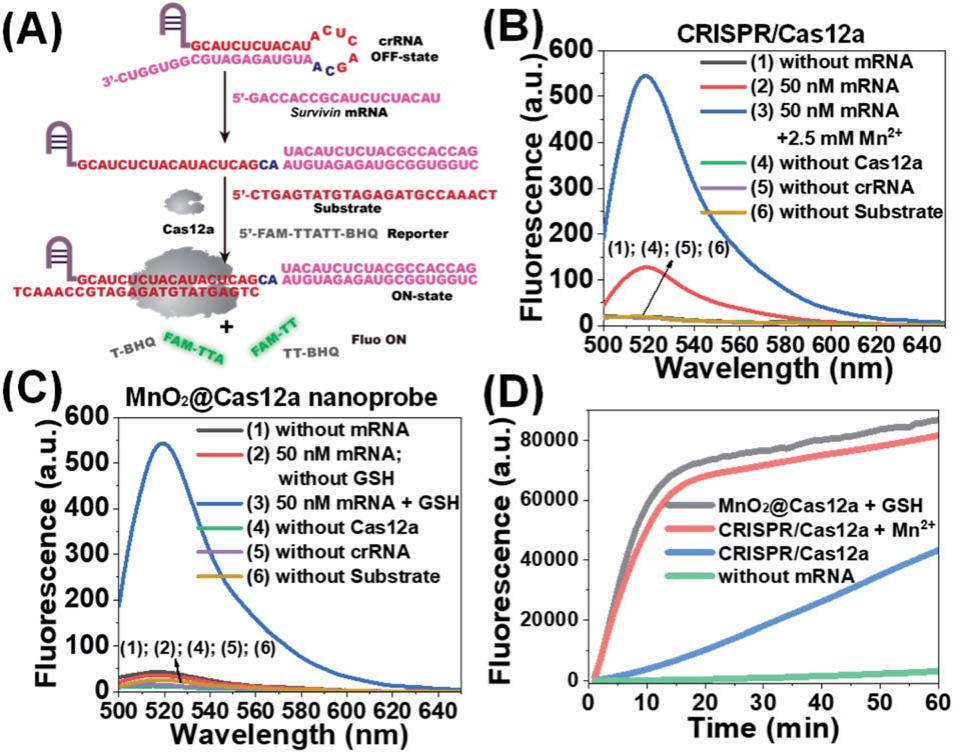
Feasibility analysis of the proposed MnO_2_/Cas12a-based sensing strategy. (A) Working mechanism of the CRISPR/Cas12a system for *survivin* mRNA detection. (B) Fluorescence spectra of CRISPR/Cas12a-based sensing systems under different conditions. (C) Fluorescence spectra of MnO_2_/Cas12a nanoprobe-based sensing systems under different conditions. (D) Fluorescence–time plots given by different sensing systems.

Before testing the biosensing performance of the MnO_2_/Cas12a nanoprobe, the target-responsive capability of the free CRISPR/Cas12a-based sensing system was verified first. Fig. 2A and S2† illustrate the working mechanism of the CRISPR/Cas12a-based sensing system. To realize the target mRNA-specific response, the crRNA was engineered to form a hairpin structure. Since the target mRNA recognition region (pink) and substrate-binding region (red) are both locked in the hairpin, the whole CRISPR/Cas12a system is in the “OFF” state. In the presence of target mRNA, the hairpin is opened through the toehold-mediated strand displacement reaction, releasing the substrate-binding region, which can then bind to the substrate strand, thus activating the *trans*-cleavage activity of CRISPR/Cas12a. As a result, the CRISPR/Cas12a system is switched from the “OFF” to “ON” state, leading to the cleavage of the fluorophore/quencher (FAM/BHQ)-labeled reporter strand and the recovery of FAM fluorescence. If Mn^2+^ is added to the system, Mn^2+^-accelerated target mRNA assay can be realized.

*Survivin* mRNA was used as the representative target mRNA to verify the feasibility of the proposed CRISPR/Cas12a-based sensing strategy. *Survivin* is an anti-apoptotic protein that can inhibit cell apoptosis and regulate cell function,^29,30^ and *survivin* detection can provide important information for the diagnosis and prognosis of malignant tumors.^31^ As shown in Fig. 2B, without *survivin* mRNA, the sensing system is in the “OFF” state, giving a very low background fluorescence. *Survivin* mRNA could activate the *trans*-cleavage activity of CRISPR/Cas12a, giving increased FAM fluorescence. The addition of Mn^2+^ resulted in the further increase of the fluorescence to a much higher level. These results, together with those from polyacrylamide gel electrophoresis (PAGE) analysis (Fig. S3†), clearly demonstrate the feasibility of the proposed sensing strategy and highlight the sensitizing effect of Mn^2+^ on CRISPR/Cas12a. Very low fluorescence was given by other negative controls lacking Cas12a, crRNA or the substrate, suggesting that these components are indispensable and must be simultaneously delivered into living cells for biosensing assay.

Different from the free CRISPR/Cas12a system, our MnO_2_@Cas12a nanoprobe gave no response to *survivin* mRNA without the help of GSH, whose fluorescence was comparable to that of the negative controls, suggesting that CRISPR/Cas12a could not work after assembly with MnO_2_ nanosheets (Fig. 2C). Such passivation of CRISPR/Cas12a is popular since it can assure the safe and efficient transportation of the CRISPR/Cas12a system to target cells. In the presence of GSH, however, a significant increase of fluorescence, which was comparable to that of the above Mn^2+^-accelerated CRISPR/Cas12a system, was given by the MnO_2_@Cas12a nanoprobe. This result is consistent with our design. That is, the decomposition of MnO_2_ nanosheets by GSH releases the CRISPR/Cas12a system, and the produced Mn^2+^ promotes the subsequent fluorescence response of CRISPR/Cas12a to *survivin* mRNA. By monitoring the time-dependent fluorescence change (Fig. 2D), it could be found that free CRISPR/Cas12a showed the slowest fluorescence response to *survivin* mRNA, and the slow fluorescence growth was continued even after 60 min. In contrast, greatly accelerated fluorescence increase was given by Mn^2+^-assisted CRISPR/Cas12a and the GSH-assisted MnO_2_@Cas12a nanoprobe, and 10 min reaction could increase the fluorescence to very high levels. These results indicate that Mn^2+^ can greatly accelerate the fluorescence response of CRISPR/Cas12a, thus achieving the fast analysis of target mRNA. The combination of MnO_2_ nanosheets and GSH can play the same role as Mn^2+^.

Since the conversion of MnO_2_ to Mn^2+^ is a crucial step, the decomposition behavior of MnO_2_ nanosheets induced by GSH was investigated. As shown in Fig. S4A,† after treatment of 10 μg mL^−1^ MnO_2_ nanosheets with 1 mM GSH, the characteristic absorption band of MnO_2_ nanosheets at 380 nm almost disappeared, indicating that the MnO_2_ nanosheets were completely destroyed by GSH. The decomposition of MnO_2_ nanosheets was so fast that the reaction could be completed within 3 min. TEM images also verified this process (Fig. S4B†). Considering that the concentration of GSH in cancer cells is about 1–10 mM,^32,33^ it can be assured that MnO_2_ nanosheets will be completely decomposed in cells, thus achieving the release of the CRISPR/Cas12a system. In contrast, negligible spectral changes were observed for MnO_2_ nanosheets when the GSH concentration was lower than 20 μM, suggesting that our MnO_2_@Cas12a nanoprobe will remain stable in human plasma and the extracellular matrix (2–20 μM GSH), which is beneficial for the highly efficient delivery of the MnO_2_/Cas12a nanoprobe to target cells.

### *Survivin* mRNA-sensing performance in solution

**Fig. 3:**
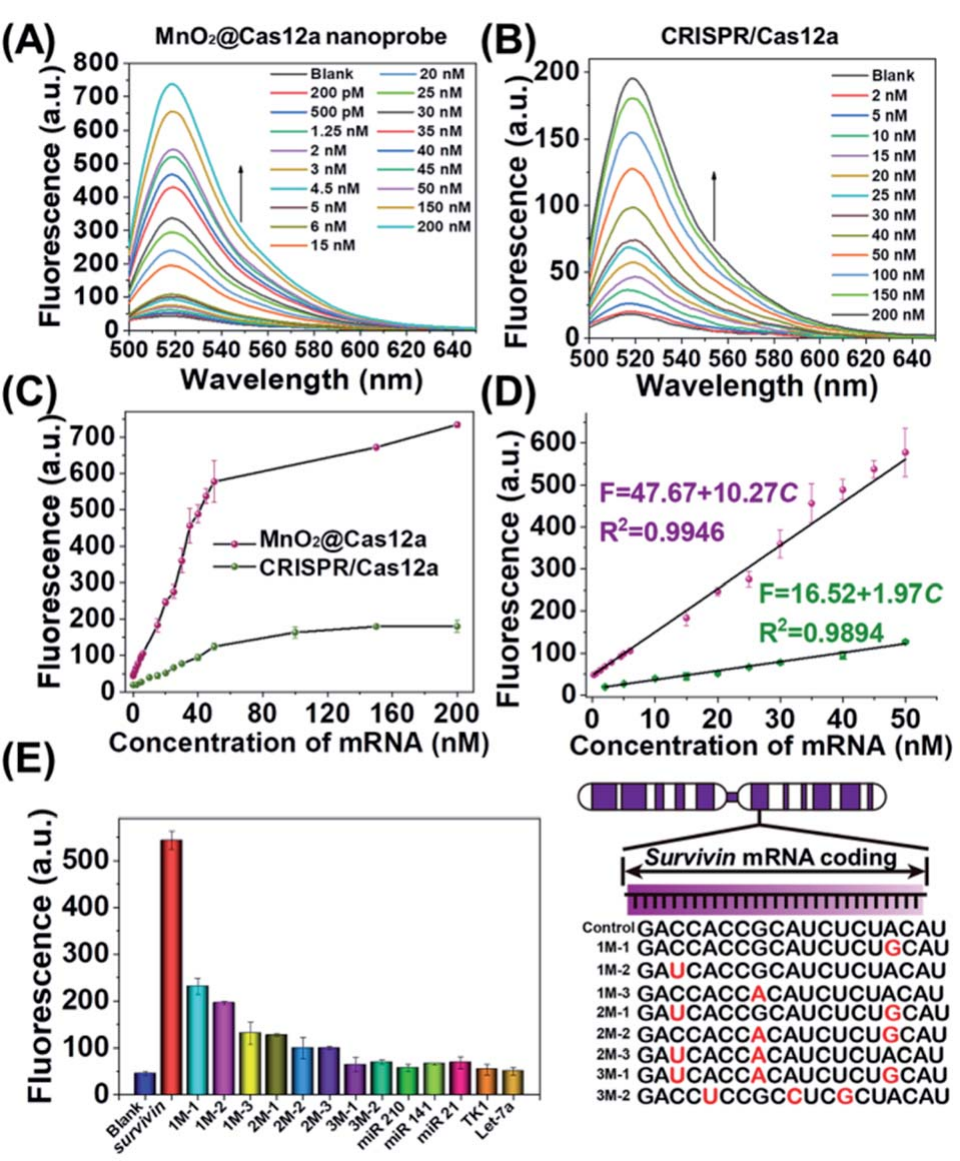
*Survivin* mRNA quantitation in solution. (A and B) Fluorescence spectra of (A) MnO_2_@Cas12a nanoprobe and (B) CRISPR/Cas12a-based sensing systems in the presence of different concentrations of *survivin* mRNA. (C) *Survivin* mRNA concentration-dependent changes in the fluorescence intensity at 520 nm given by the two sensing systems. (D) The linear relationships between fluorescence and the mRNA concentration for the two sensing systems. (E) Selectivity of the MnO_2_@Cas12a-based sensing system. The concentration of all tested RNAs was 50 nM. The sequences of all tested RNAs are given in Table S1.† Error bars represent the standard deviation of three repetitive experiments.

Before live-cell applications, the target mRNA-sensing capability of the MnO_2_/Cas12a nanoprobe was first tested in solution. To give the best sensing performance, some critical experimental conditions were optimized (Fig. S5–S11, Table S2†). Through repeated optimization, the optimal reaction conditions were obtained: 100 nM substrate with 18-nucleotide length, 2.5 μg mL^−1^ MnO_2_ nanosheets/0.5 mM GSH, 100 nM crRNA, 50 nM Cas12a, 100 nM reporter, and 10 min for CRISPR/Cas12a-catalyzed reporter cleavage. Under the optimized conditions, the fluorescence of the sensing system continuously increased with the target mRNA concentration (Fig. 3A and C), and a linear relationship (*R*^2^ = 0.9946) was observed in the mRNA concentration range of 200 pM–50 nM (Fig. 3D). Based on the 3*σ*/slope criterion (*σ* is the standard deviation of 10 blank samples, the slope is the slope of the standard curve), the detection limit was calculated to be 67 pM, which is equal to or lower than other reported results (Table S3†). The whole detection could be completed within 15 min, making simple and rapid target detection possible.

To highlight the contribution of Mn^2+^, which is produced by GSH-induced decomposition of MnO_2_ nanosheets, the mRNA-sensing performance of free CRISPR/Cas12a was investigated for comparison. As mentioned above, without the help of Mn^2+^, CRISPR/Cas showed a much slower fluorescence response to target mRNA. Meanwhile, the fluorescence signal output was greatly decreased (Fig. 3B). The linear detection range was 2–50 nM, and the detection limit was calculated to be about 650 pM, which was nearly 1 order higher than that given by the MnO_2_/Cas12a nanoprobe.

Good detection selectivity was given by the MnO_2_@Cas12a nanoprobe (Fig. 3E). The mRNAs with single-base mismatches could be easily distinguished from target mRNA even when the mismatched base was near to the two ends of the recognition region. The mRNAs with three mismatched bases and the RNAs (mRNAs and microRNAs) with unrelated sequences showed no any detectable fluorescence changes compared to the blank control. In general, the above experimental results demonstrate that our MnO_2_/Cas12a-based sensing platform is very feasible for rapid, sensitive and selective detection of target mRNA in an easy way.

### *Survivin* mRNA biosensing in living cells

Toxicity has always been an important factor in the design of probes for live-cell applications. The potential cytotoxicity of the MnO_2_@Cas12a nanoprobe to three different cells (HeLa, MCF-7 and HEK293 cells) was evaluated by cell viability analysis. After 24 hours of incubation with different concentrations of the MnO_2_@Cas12a nanoprobe (up to 40 μg mL^−1^ MnO_2_ nanosheets), the viabilities of the three kinds of cells were all maintained above 80% (Fig. S12†), indicating that the nanoprobe shows low toxicity at working concentrations. Besides, excess Mn^2+^ can be effluxed by transport membrane proteins, which benefits the clearance of excess Mn^2+^ after live-cell biosensing analysis.^34^

Having demonstrated the low toxicity of the MnO_2_@Cas12a nanoprobe, we next investigated its feasibility for *survivin* mRNA-sensing in living cells. Previous literature reported that MnO_2_ nanosheets could enter cells through scavenger receptor-mediated phagocytosis.^35^ Using 10 μg mL^−1^ MnO_2_ nanosheets as the carrier, all components of the CRISPR/Cas12a system, including 150 pmol of substrate, 150 pmol of crRNA, 150 pmol of reporter, and 80 pmol of Cas12a could be synchronously delivered into living cells. As shown in Fig. S13,† only the MnO_2_@Cas12a nanoprobe-based system, in which all components were included, gave very bright green fluorescence in cells. Very weak background fluorescence was given by the combinations of MnO_2_ nanosheets with reporter, reporter/substrate, or reporter/substrate/crRNA. These results were consistent with those of sensing assay in solution, thus indicating that the bright FAM fluorescence given by the MnO_2_@Cas12a nanoprobe was related with the activation of CRISPR/Cas12a *trans*-cleavage activity in cells, but not caused by unspecific degradation of the reporter strand. After monitoring the changes in fluorescence intensity as a function of incubation time (0.5–4 hours), it could be found that the green fluorescence of FAM gradually increased with incubation time, and approached equilibrium at 2 h (Fig. S14†). Therefore, 2 h incubation time was used for subsequent live-cell experiments.

**Fig. 4:**
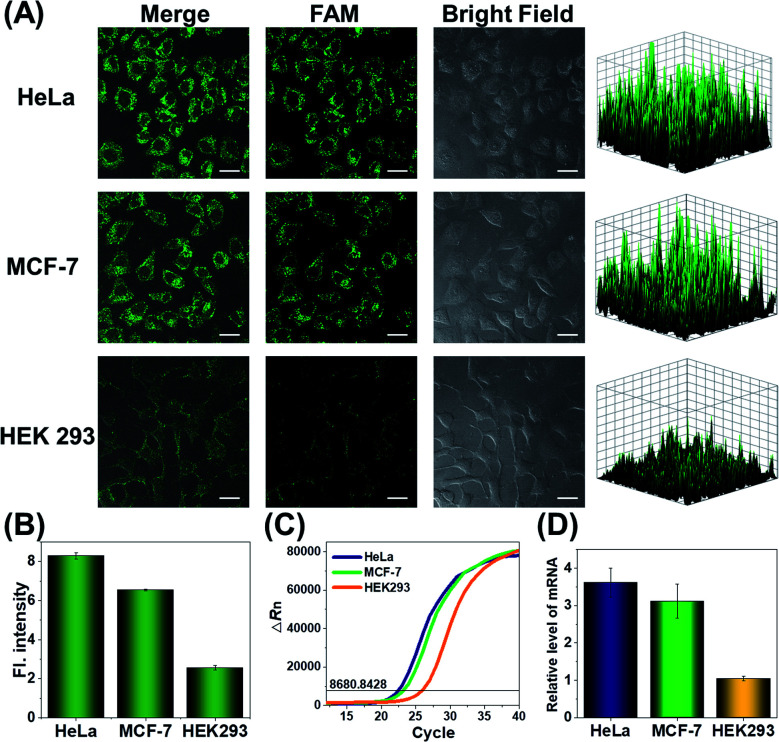
(A) Confocal laser scanning microscopy (CLSM) analysis of *survivin* mRNA in HeLa, MCF-7, and HEK293 cells. Scale bar is 25 μm. The right-hand figures show the fluorescence intensities of corresponding fluorescence images. (B) Corresponding histogram of fluorescence intensity in the above cells. Data represent means ± SD (91, 92, and 95 cells were measured from top to bottom). (C) Real-time fluorescence curves obtained in qRT-PCR analysis. The black line indicates the threshold, and the number on the line indicates the threshold value. (D) Relative expression levels of *survivin* mRNA determined by RT-PCR. Error bars are the standard deviations of three repeated determinations.

Since the *survivin* mRNA expression dynamically changes in different cell lines, we selected three cell lines with different *survivin* mRNA expressions for live-cell biosensing analysis. HeLa and MCF-7 cell lines, which are known to express relatively high levels of *survivin* mRNA, were selected as positive cells, while the HEK293 cell line with low *survivin* mRNA expression was selected as a negative control.^36,37^ As shown in Fig. 4A, HeLa and MCF-7 cells showed much stronger FAM fluorescence than HEK293 cells, which is consistent with the reported *survivin* mRNA expression levels in these cells,^38^ thus suggesting that the fluorescence is really caused by *survivin* mRNA-induced activation of CRISPR/Cas12a. To further demonstrate the close relationship between the fluorescence signal and *survivin* mRNA expression, the expression levels of *survivin* mRNA in different cell lines were analyzed by using quantitative reverse transcriptase-polymerase chain reaction (qRT-PCR) (Fig. 4C). Highly consistent results were given by our analysis and qRT-PCR (Fig. 4B and D), suggesting that our CRISPR/Cas12a nanoprobe could be used to discriminate different cell lines according to their mRNA expression levels.

### Analysis of intracellular *survivin* mRNA expression fluctuation

**Fig. 5:**
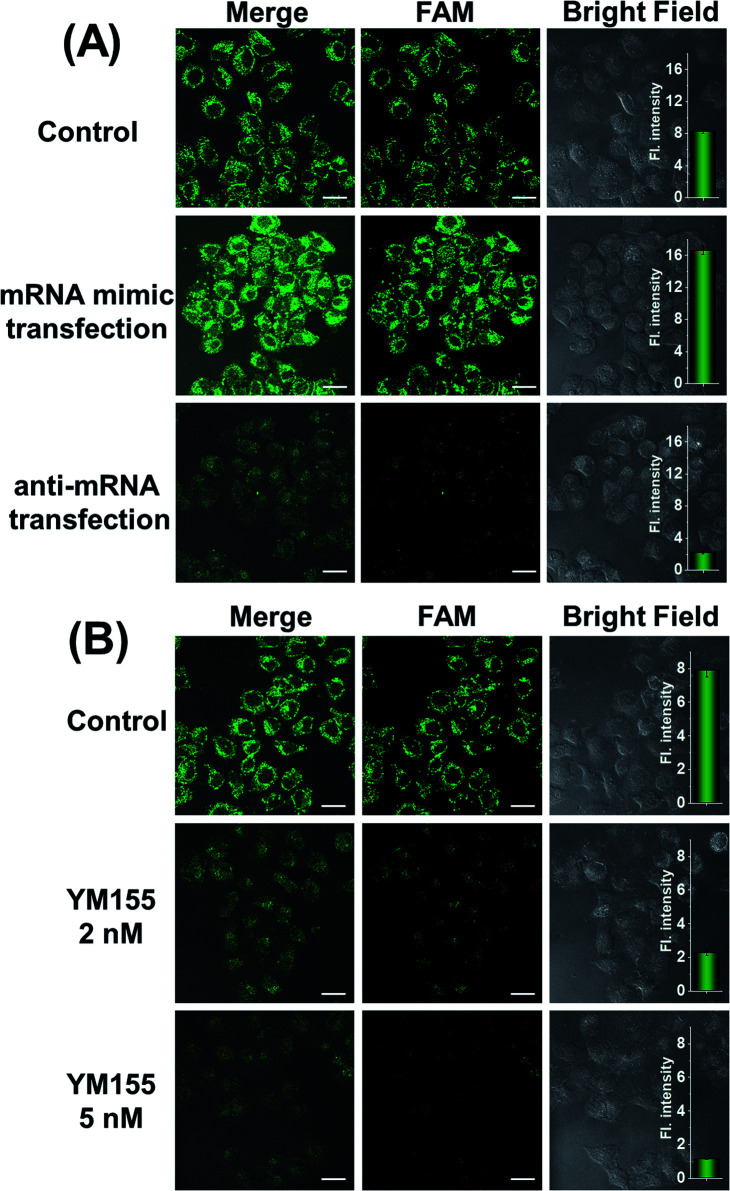
(A) CLSM images of HeLa cells transfected with the mRNA mimic or anti-mRNA. (B) CLSM images of HeLa cells treated with YM 155 (0 nM, 2 nM and 5 nM). Scale bar is 25 μm. The insets show the quantified normalized fluorescence intensities from these CLSM images using Image-J software. Data represent means ± SD (97, 95, 93, 96, 91, and 96 cells were measured from top to bottom).

At different stages of tumorigenesis, even in the same cancer cell, mRNA expression levels may change, so it is necessary to use the MnO_2_@Cas12a nanoprobe to detect the changes in *survivin* mRNA expression levels. HeLa cells were divided into three groups: the first group was control without any pretreatment; the second group was transfected with a *survivin* mRNA mimic (Table S1†) to increase the mRNA expression; the third group was transfected with an anti-mRNA strand (Table S1†) to reduce the mRNA expression level. The three groups were respectively incubated with the MnO_2_@Cas12a nanoprobe for 2 hours. As shown in Fig. 5A, compared with the control group, a brighter green fluorescence was observed in the mRNA mimic-transfected group, and a much weaker fluorescence was given by the group transfected with anti-mRNA. These results indicate that the MnO_2_@Cas12a nanoprobe can accurately probe different expression levels of target mRNA in living cells. To give a further illustration, YM155, a *survivin* inhibitor, was used to treat HeLa cells to down-regulate intracellular *survivin* mRNA.^39^ Analysis of YM155-treated cells using the MnO_2_@Cas12a nanoprobe clearly showed that the fluorescence intensity decreased with increasing YM155 concentration (Fig. 5B), thus confirming that the fluorescence is indeed closely related to the content of *survivin* mRNA.

### Comparison with commercial liposome carriers

**Fig. 6:**
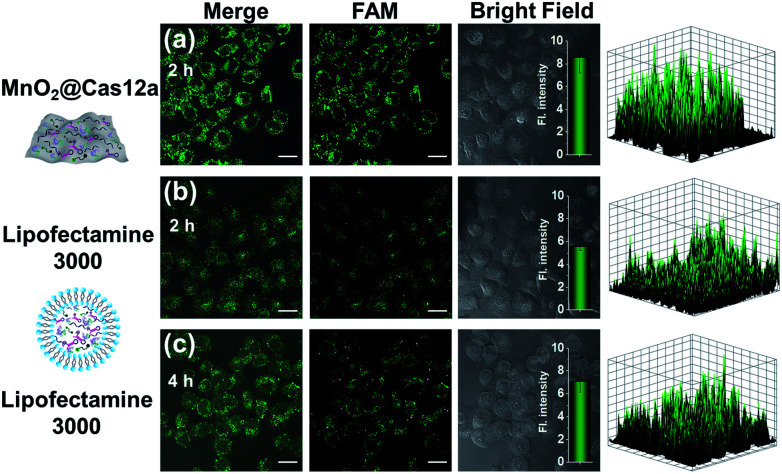
Intracellular *survivin* mRNA analysis using (a) MnO_2_ nanosheets or (b and c) Lipofectamine 3000 as the carrier of CRISPR/Cas12a. The incubation time was labeled in the figures. Scale bar is 25 μm. The insets show the normalized fluorescence intensities of the cell images. Data represent means ± SD (90, 89, and 94 cells were measured from top to bottom). The right-hand figures show the fluorescence intensities of corresponding fluorescence images.

To make an intuitive evaluation of the performance of MnO_2_ nanosheets as the CRISPR/Cas12a carrier for live-cell application, commercial liposome was selected as the carrier of CRISPR/Cas12a for comparison. As shown in Fig. 6, commercial liposome carriers (*e.g.*, Lipofectamine 3000) could also deliver substrate/reporter/crRNA/Cas12a into cells. However, different from MnO_2_@Cas12a nanoprobe-treated cells that gave bright images at 2 h, the cells treated with Lipofectamine 3000 carrier exhibited much weaker fluorescence at 2 h. Even when the incubation time was prolonged to 4 h, the fluorescence intensity in Lipofectamine 3000-treated cells was still lower than that given by the cells with 2 h MnO_2_@Cas12a treatment. These results suggest that MnO_2_ nanosheets can offer not only a time advantage but also a stronger signal output than commercial liposome carriers. The better performance of MnO_2_ nanosheets than commercial liposomes might be attributed to the following reasons: (1) highly efficient delivery of the CRISPR/Cas12a system into cells. Since the extracellular GSH concentration (<20 μM) is much lower than that in cells (1–10 mM), the MnO_2_@Cas12a nanoprobe remains stable before entering cells, thus making highly efficient internalization by cells possible. (2) Rapid release of the CRISPR/Cas12a system in cells. Due to the high concentration of GSH in cells, MnO_2_ nanosheets might be completely decomposed in a few minutes. Therefore, the CRISPR/Cas12a system can be rapidly released from MnO_2_@Cas12a to perform target mRNA-specific analysis. (3) Mn^2+^-accelerated CRISPR/Cas12a *trans*-cleavage activity. Intracellular decomposition of MnO_2_ nanosheets produces Mn^2+^, which can then be used as the accelerator to promote CRISPR/Cas12a-catalyzed cleavage of the reporter strand, giving a significantly increased fluorescence signal in a short time.

**Fig. 7:**
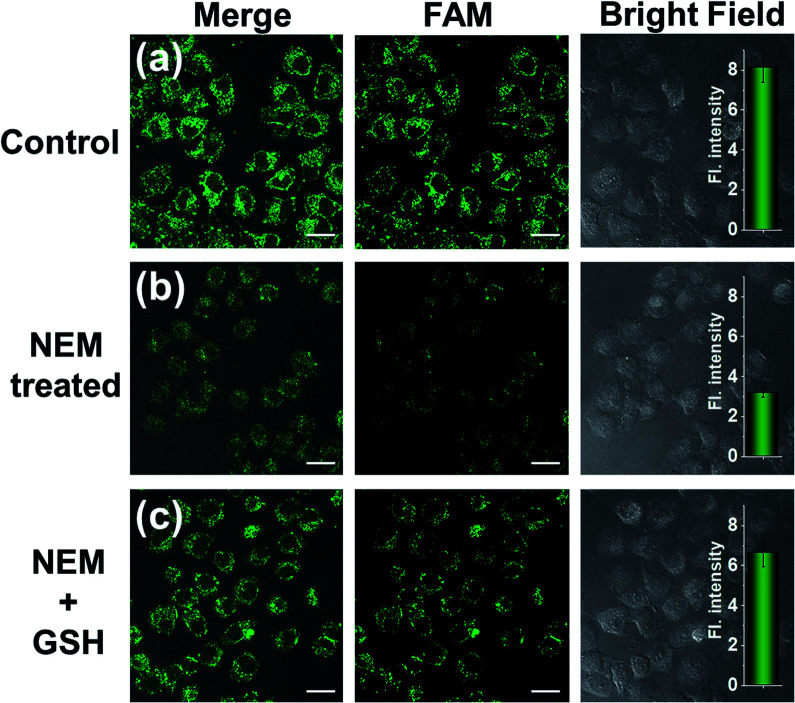
*Survivin* mRNA expression level detection in different HeLa cells using the MnO_2_@Cas12a nanoprobe. (a) HeLa cells without any treatment; (b) HeLa cells were pretreated with 1 mM NEM; (c) HeLa cells were pretreated with 1 mM NEM and then with 500 μM GSH. Scale bar is 25 μm. The insets show the normalized fluorescence intensities determined from the confocal images. Data represent means ± SD (96, 90, and 92 cells were measured from top to bottom).

GSH-induced intracellular decomposition of MnO_2_ nanosheets not only rapidly releases the CRISPR/Cas12a system, but also produces Mn^2+^ to work as the accelerator of CRISPR/Cas12a. To verify the dependence of the MnO_2_@Cas12a nanoprobe on intracellular GSH, HeLa cells were treated with *N*-ethylmaleimide (NEM) to eliminate endogenous thiols including GSH.^40^ Compared to the control without NEM treatment, NEM-treated cells displayed very weak fluorescence after incubation with MnO_2_@Cas12a for 2 h (Fig. 7). In contrast, when NEM-treated cells were further incubated with 500 μM GSH for another 20 min to supplement intracellular GSH, the fluorescence was greatly recovered. These results highlight the GSH dependence of the MnO_2_@Cas12a nanoprobe, which is consistent with the proposed working mechanism.

## Conclusion

In summary, we have demonstrated that MnO_2_ nanosheets are a good carrier to promote the applications of CRISPR/Cas12a in living cells. Herein, MnO_2_ nanosheets play two roles: a carrier that delivers CRISPR/Cas12a into cells and an accelerator that provides Mn^2+^ to promote the CRISPR/Cas12a-catalyzed *trans*-cleavage reaction. Compared to other carriers, MnO_2_ nanosheets show the following two distinct advantages as the carrier of CRISPR/Cas12a: (1) easy CRISPR/Cas12a-loading operation. Through a simple mixing operation, all components of the CRISPR/Cas12a system can be easily loaded on MnO_2_ nanosheets, thus achieving synchronous delivery of these components into cells. (2) Improved biosensing performance in living cells. By comparing Lipofectamine 3000, MnO_2_ nanosheets could produce much brighter fluorescence images in a shorter time due to highly efficient delivery, rapid intracellular release and the accelerated signal output reaction. In this work, we used *survivin* mRNA as a representative target to test the feasibility of the MnO_2_/Cas12a nanoprobe for live-cell biosensing applications. By simply changing the CRISPR/Cas12a-based sensor design, MnO_2_/Cas12a nanoprobes could be easily extended to the intracellular sensing analysis of various targets, including mRNA, microRNA, enzymes, proteins and other biological molecules. In addition, there have been several reports attempting to utilize CRISPR/Cas12a for imaging living animals.^41^ There is reason to believe that MnO_2_ nanosheets can serve as biocompatible carriers to facilitate the applications of these CRISPR/Cas systems in living cells and even *in vivo*.

## Data availability

Experimental details and additional data can be found in the attached ESI.†

## Author contributions

Dong-Xia Wang: conceptualization, methodology, investigation, writing-original draft. Ya-Xin Wang: visualization, investigation. Jing Wang: investigation. Jia-Yi Ma: investigation. Bo Liu: validation. An-Na Tang: supervision and reviewing. De-Ming Kong: supervision, reviewing and editing.

## Conflicts of interest

There are no conflicts to declare.

## Supplementary Material

SC-013-D1SC06383A-s001
