# Supplementary material for: MnO2 nanosheets as a carrier and accelerator for improved live-cell biosensing application of CRISPR/Cas12a
Source: Chem Sci. 2022 Mar 21;13(15):4364–71. doi: 10.1039/d1sc06383a (PMC9007066; doi:10.1039/d1sc06383a)
Supplement: SC-013-D1SC06383A-s001 [file SC-013-D1SC06383A-s001.pdf]

Electronic Supplementary Material (ESI) for Chemical Science.
This journal is © The Royal Society of Chemistry 2022

Supporting Information for

MnO_2 Nanosheets as Carrier and Accelerator for Improved Live-Cell Biosensing Application of CRISPR/Cas12a

Dong-Xia Wang[a], Ya-Xin Wang[a,b], Jing Wang[a], Jia-Yi Ma[a], Bo Liu[a], An-Na Tang[a], De-Ming Kong[a,*]

a. State Key Laboratory of Medicinal Chemical Biology, Tianjin Key Laboratory of Biosensing and Molecular Recognition, Research Centre for Analytical Sciences, College of Chemistry, Nankai University, Tianjin 300071, P. R. China

b. School of Pharmacy, Binzhou Medical University, Yantai, Shandong, 264003, PR China

E-mail: kongdem@nankai.edu.cn

Table of Contents

Experimental section……….…………………..S-1

UV-Vis spectra of CRISPR/Cas12a systems before and after assembly with MnO_2 nanosheets…………….......................….........................S-6

Working mechanism of CRISPR/Cas12a-based sensing strategy………………S-7

PAGE characterization of CRISPR/Cas12a-based mRNA-sensing strategy…..S-8

Decomposition of MnO_2 nanosheets by GSH……………………………..……S-9

Optimization of MnO_2@Cas12a-based mRNA-sensing in solution………......S-10

Comparison of our detection method with other reported ones……………..S-14

Cytotoxicity evaluation of MnO_2@Cas12a nanoprobe………………………S-15

Feasibility of MnO_2@Cas12a for intracellular mRNA-imaging………………S-16

Optimization of incubation time for cell imaging……………….…………S-17

Reference………...S-18

1. Experimental section

1.1. Materials and reagents

All DNA oligonucleotides (Table S1) were synthesized and purified by Sangon Biotech. Co. Ltd. (Shanghai, China). RNA oligonucleotides were purchased from GenScript. Biotech. Co. Ltd. (Nanjing, China). $MnCl_2 \cdot 4H_2O$ and tetramethylammonium hydroxide (TMA•OH) were purchased from Alfa Aesar (China). Lba Cas12a (cpf1) was obtained from Tolo Biotech., 10 × 2.1 buffer (500 mM NaCl, 100 mM Tris-HCl, 100 mM $MgCl_2$, 100 μg/ml BSA, pH 7.9) was purchased from New England Biolabs (Beijing, China). Glutathione (GSH), *N*-ethylmaleimide (NEM) and YM155 were bought from Sigma-Aldrich Chemical Co. Ltd. Lipofectamine 3000 was purchased from Invitrogen. Dulbecco's modified Eagle's medium (DMEM) supplemented with 10% fetal bovine serum (FBS), penicillin (100 U/mL), streptomycin (100 μg/mL) and amphotericin B (0.25 μg/mL) was used for cell culture. Trypsin/EDTA (0.25%) was obtained from Gibco (Thermo Fisher Scientific). All other reagents were analytical grade and directly used without further purification. Ultrapure water (resistance ≥ 18.2 MΩ·cm) was used throughout the experiments.

1.2. Apparatus

Fluorescence spectra were recorded on a Shimadzu RF-5301 PC fluorescence spectrometer (Shimadzu Ltd., Japan) using 488 nm as the excitation wavelength. Excitation and emission slits were both set at 5.0 nm. Time-dependent fluorescence changes were recorded on a commercial StepOnePlus™ Real-Time PCR instrument (Applied Biosystems, USA). Ultraviolet-visible (UV-Vis) spectra were recorded on the TU-1901 spectrophotometer (Persee, Beijing). Dynamic light scattering (DLS) experiments were performed on the Malvern Zetasizer Nano ZS90 (Malvern Instruments, Ltd., Worcestershire, UK) at room temperature. Transmission electron microscopy (TEM) images were measured on HITACHI HT7700 Exalens (Japan). Fluorescence images of cells were measured on a confocal laser scanning fluorescence microscope setup (Nikon A+) equipped with a 60 × oil objective lens.

Table S1. Sequences of the oligonucleotides used in this work.

Oligonucleotide		Sequences (5' to 3')
mRNA detection	crRNA	UAAUUUCUACUAAGUGUAGAU GCA UCU CUA CAU ACU CAG CA AUG UAG AGA UGC GGU GGU C
	Substrate	CTG AGT ATG TAG AGA TGC CAAACT
	Survivin mRNA	GAC CAC CGC AUC UCU ACA U
	Reporter	FAM-TTATT-BHQ1
	FAM-ssDNA	FAM-TCATGTTTGTTTGTTGGCCCCCCTTCTTTCTTA
	1M-1	GAC CAC CAC AUC UCU ACA U
	1M-2	GAU CAC CGC AUC UCU ACA U
	1M-3	GAC CAC CGC AUC UCU GCA U
	2M-1	GAC CAC CAC AUC UCU GCA U
	2M-2	GAU CAC CGC AUC UCU GCA U
	2M-3	GAU CAC CAC AUC UCU ACA U
	3M-1	GAU CAC CAC AUC UCU GCA U
	3M-2	GAC CUC CGC CUC GCU ACA U
	miR21	UAG CUU AUC AGA CUG AUG UUG A
	Let-7a	UGA GGU AGU AGG UUG UAU AGU U
	miR210	CUG UGC GUG UGA CAG CGG CUG A
	miR141	UAA CAC UGU CUG GUA AAG AUG G
	TK1	UGAUCAAGUAUGCCAAAGACACUCGCUACA

1.3. Preparation of MnO_2 nanosheets

MnO_2 nanosheets were prepared on the basis of the previous method of our research group.[1] Briefly, 0.6 M TMA•OH and 3 wt% H_2O_2 were added in 10 mL of 0.3 M $MnCl_2$ solution within 15 s, the total volume was 20 mL. The solution turned dark brown immediately, and the dark brown suspension was stirred vigorously in the open air overnight at room temperature. The prepared bulk MnO_2 was centrifuged at 2000 rpm for 10 min, and then washed with a large amount of distilled water and ethanol. After that, the MnO_2 was dried in an oven at 60 ºC. To prepare MnO_2 nanosheets, 20 mg of MnO_2 was dispersed in 20 mL of distilled water and sonicated for 10 h (ShuMei Ultrasonic Cleaner KQ-300DE; KunShan, China).

1.4. *Survivin* mRNA-sensing performance in solution

2.5 μg/mL MnO_2 nanosheets was incubated with 0.5 mM GSH. 2 min latter, 2.1 buffer (1×), Substrate (100 nM), Cas12a (50 nM), crRNA (100 nM), Reporter (100 nM), and differenct concentrations of *Survivin* mRNA were added to a total volume of 100 μL. The mixture was incubated at 37 ºC for 10 min, and its fluorescence spectrum was recorded on a Shimadzu RF-5301 PC fluorescence spectrometer (Shimadzu Ltd., Japan) with an excitation wavelength of 488 nm. Excitation and emission slits were both set at 5.0 nm.

1.5. Non-denaturing polyacrylamide gel electrophoresis (PAGE) analysis

To verify target mRNA-induced activation of CRIPSR/Cas12a, 20 μL of reaction samples containing 1 × 2.1 buffer, 2.5 μM of Substrate, 2.5 μM of crRNA and 2.5 μM of *Survivin* mRNA were prepared. Then, 2 μL 6 × Super GelRed Prestain Loading Buffer (US EVERBRIGHT INC.) was added and sufficiently mixed. The samples were analyzed by 10% PAGE in 1 × TAE buffer at a 120 V constant voltage for 50 min. The gel was visualized using a Gel Image System (Amersham Biosciences).

To characterize single-stranded DNA (ssDNA) cleavage by activated CRIPSR/Cas12a, FAM-ssDNA with 33 nucleotides (Table S1) was used as the probe for non-denaturing PAGE analysis. The experimental operations were the same as above except that Cas12a (500 nM) and FAM-ssDNA (2.5 μM) were added. The gel was photographed directly via the fluorescence of the fluorophore FAM.

1.6. Preparation of MnO_2@Cas12a nanoprobe

MnO_2@Cas12a nanoprobe was prepared by mixing 50 μL of MnO_2 nanosheets (100 μg/mL), 1.5 μL of Substrate (50 μM), 1.5 μL of crRNA (50 μM) and 1.5 μL of Reporter (50 μM) for 20 min, followed by addition of 10 μL of Tris-HAc buffer (100 mM, pH 7.4). After that, 4 μL cas12a (10 μM) was added into above mixture and incubated for another 10 min at room temperature. As-prepared MnO_2@Cas12a could be directly used for subsequent cell experiments.

1.7. Cell culture and cytotoxicity evaluation of MnO_2@Cas12a

HeLa, MCF-7 and HEK293 cells were obtained from ATCC (American Type Culture Collection, Manassas, VA, USA) and cultured in DMEM medium, which was supplemented with 10% FBS, penicillin (100 U/mL), streptomycin (100 μg/mL), amphotericin B (0.25 μg/mL), in a humidified atmosphere containing 5% CO_2 at 37 °C. The cytotoxicity of MnO_2@Cas12a was evaluated using a standard cell counting kit-8 (CCK-8) assay. HeLa, MCF-7 and HEK-293 cells were seeded in a 96-well plate with a cell density of 10^4 per well. After the cells were completely adherent, different concentrations of MnO_2@Cas12a (10 μg/mL, 20 μg/mL, 30 μg/mL and 40 μg/mL of MnO_2 nanosheets) were added into the 96-well plate and then incubated with the cells for 4, 12 or 24 h. Then, 10 μL CCK-8 solution was added to each well and the absorbance value at 450 nm was measured *via* a Microplate Reader. Finally, cell viability was calculated according to the manufacturer's protocol. All the experiments were repeated at least three times.

1.8. Confocal laser scanning microscopy (CLSM) image analysis

HeLa, MCF-7 or HEK293 cells were seeded in a 15 mm confocal dish and incubated at 37 °C in 5% CO_2 for 24 h. After washing 3 times with PBS buffer, 500 μL of fresh cell growth medium supplemented with MnO_2@Cas12a nanoprobe was added. The final concentration of each component was 10 μg/mL of MnO_2 nanosheet; 150 pmol of Substrate; 80 pmol of Cas12a; 150 pmol of crRNA and 150 pmol of Reporter. After an incubation period of 2 h, 1 × PBS buffer was employed to wash the cells 3 times to remove MnO_2@Cas12a nanoprobes that were not taken up by cells. The CLSM images of the cells were observed using a NIKON A+ confocal microscope with laser excitation at 490 nm.

To perform cell imaging analysis of *Survivin* inhibitor YM155-treated cells, 2 nM or 5 nM YM155 was utilized to treat HeLa cells for 24 h at 37 °C. The cell imaging analysis were performed as metioned above.

To compare MnO_2 nanosheets with commercial liposome carriers, Lipofectamine 3000 was selected as the carrier of CRISPR/Cas12a to perform the imaging analysis of intracellular *Survivin* mRNA. Transfection assays were performed according to the

manufacturer's protocol. Briefly, after cells reached ~70 % confluence, the cells were transfected by adding 150 nM of Substrate; 80 nM of Cas12a; 150 nM of crRNA and 150 nM of Reporter using Lipofectamine 3000 (Invitrogen) in 200 µL Opti-MEM at 37 °C for 2 h or 4 h. The cells were washed three times with 1×PBS before imaging.

To verify the GSH dependence of MnO_2@Cas12a, the cells were divided into two groups. One group was incubated with *N*-ethylmaleimide (NEM) for 30 min, the other group was was incubated with *N*-ethylmaleimide (NEM) for 30 min and then with 500 µM GSH for another 20 min. Then, cell imaging analysis was conducted by using MnO_2@Cas12a nanoprobe.

1.9. RT-PCR quantification of *Survivin* mRNA expression

Total cellular RNAs were extracted from HeLa, MCF-7 and HEK293 cells (1.0 mL, 1×10^7 cells) by using Trizol total RNA extraction kit (Sangon, Shanghai). RT-PCR analysis was performed on an ABI Stepone Plus qPCR instrument using a BeyoFast™ SYBR Green One-Step qRT-PCR Kit (Beyotime, China) according to the protocol. The detailed steps were as follows: A 20 µL of reaction solution was kept at 50 °C for 30 min, then holded at 95 °C for 2 min followed by 45 cycles at 95 °C for 15 s and at 60 °C for 30 s. β-actin RNA was used as the endogenous control. The $2^{-(\Delta\Delta Ct)}$ method was utilized to calculate relative *Survivin* mRNA expression level. Primers for qPCR were:

Survivin forward primer: 5′-GCCATTAACCGCCAGATTT-3′;

Survivin reverse primer: 5′-CCTCTGCGACAGCTTATAATGG-3′;

β-actin forward primer: 5′-TAGTTGCGTTACACCCTTTCTTG-3′;

β-actin reverse primer: 5′-TCACCTTCACCGTTCCAGTTT-3′.

2. UV-Vis spectra of CRISPR/Cas12a systems before and after assembly with MnO_2 nanosheets

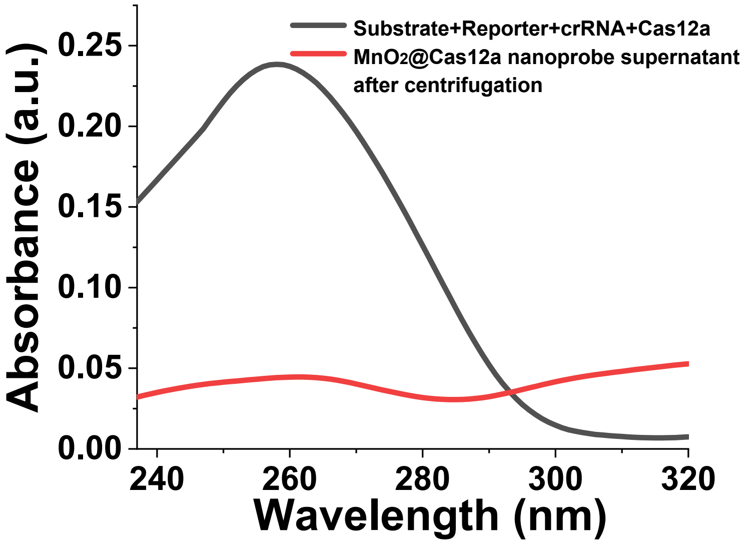

Figure S1. UV-Vis absorption spectra of Substrate+Reporter+crRNA+Cas12a mixture before and after assembly with MnO_2 nanosheets. After assembly reaction, the MnO_2@Cas12a nanoprobe was removed by high-speed centrifugation, and the UV-Vis absorption spectra of the supernatant was recorded.

3. Working mechanism of CRISPR/Cas12a-based *Survivin* mRNA-sensing strategy

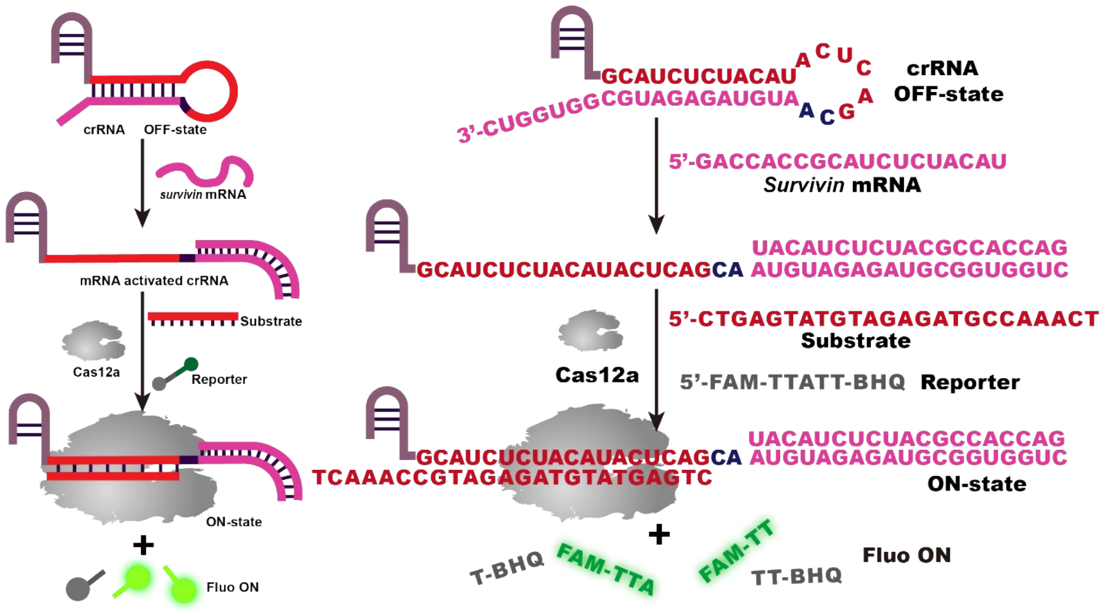

Figure S2. Working mechanism of CRISPR/Cas12a-based *Survivin* mRNA-sensing strategy. The detailed DNA and RNA sequences were given on the right.

4. PAGE characterization of CRISPR/Cas12a-based *Survivin* mRNA-sensing strategy

Polyacrylamide gel electrophoresis (PAGE) was used to verify the proposed *Survivin* mRNA-sensing strategy. First, the interactions of crRNA with *Survivin* mRNA and Substrate were investigated. As shown in Figure S2A, without *Survivin* mRNA, crRNA could not hybridize with Substrate strand, giving two separate bands corresponding to crRNA and Substrate strands, respectively (Lane 4). *Survivin* mRNA could open the hairpin structure of crRNA, releasing the binding region of Substrate strand. As a result, when Substrate, crRNA, and mRNA were all present, a bright band with much slower mobility could be clearly observed (Lane 6), indicating the formation of ternary Substrate/crRNA/mRNA complex.

To demonstrate that *Survivin* mRNA can activate the *trans*-cleavage activity of CRISPR/Cas12a to perform the cleavage of single-stranded DNA (ssDNA) probe, a fluorophore-labeled ssDNA probe (FAM-ssDNA, Table S1) with 33-nucleotide length was selected for PAGE analysis of cleavage reaction. As shown in Figure S2B, only when Substrate, crRNA, *Survivin* mRNA and Cas12a were all present, obvious cleavage of FAM-ssDNA could be observed (Lane 5). On the contrary, FAM-ssDNA would keep intact if anyone was missing.

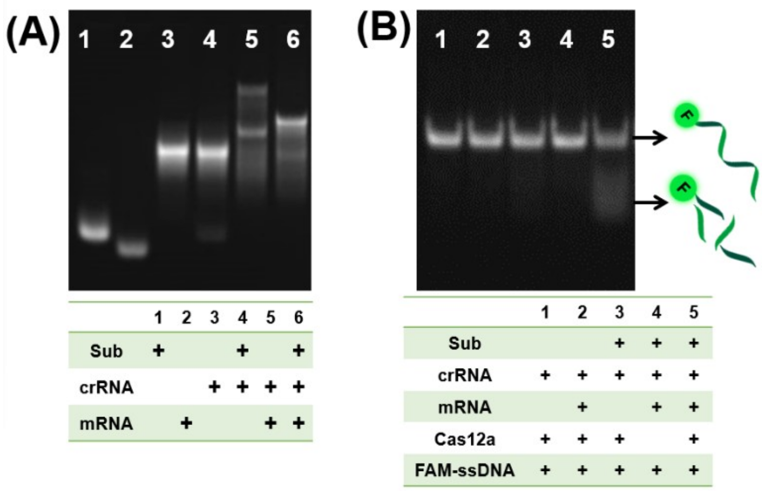

Figure S3. (A) Non-denaturing PAGE characterization on the interactions of crRNA with *Survivin* mRNA and Substrate. (B) Non-denaturing PAGE characterization of ssDNA probe cleavage by activated CRISPR/Cas12a.

5. Decomposition of MnO_2 nanosheets by GSH

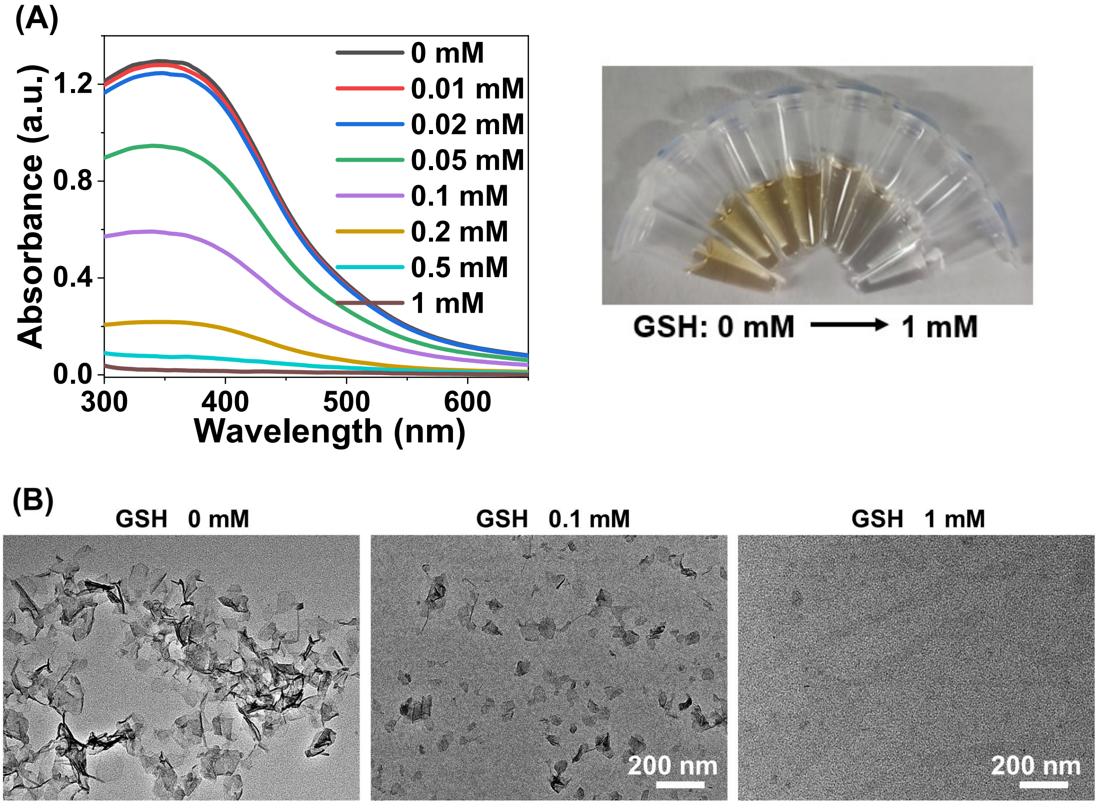

Figure S4. (A) Ultraviolet-visible spectra of 10 μg/mL MnO_2 nanosheets after treatment with different concentrations of GSH. Corresponding images of the solutions were shown on the right hand. With the decomposition of MnO_2 nanosheets, the solution color was changed from brown to transparent. (B) TEM images of MnO_2 nanosheets after treatment with different concentrations of GSH.

6. Optimization of MnO_2@Cas12a-based mRNA-sensing in solution

6.1. Substrate type

Table S2. Different substrate strands tested in this work.

Oligonucleotide		Sequences (5' to 3')
Substrate strand	M1	AG CTG AGT ATG TAG AGA TGC CAAACT
	M2	TA CTG AGT ATG TAG AGA TGC CAAACT
	M3	TG ATG AGT ATG TAG AGA TGC CAAACT
	M4	TG ACG AGT ATG TAG AGA TGC CAAACT
	M5	TG CTC AGT ATG TAG AGA TGC CAAACT
	M6	TG CTG ACT ATG TAG AGA TGC CAAACT
	M7	TG CTG TGT ATG TAG AGA TGC CAAACT
	M8	TG CTG AGC ATG TAG AGA TGC CAAACT
	M9	TG CTG AGT AGG TAG AGA TGC CAAACT
	M10	TG CTG AGT ATG CAG AGA TGC CAAACT
	M11	TG CTG AGT ATG TAC AGA TGC CAAACT
	M12	TG CTG AGT ATG TAG ACA TGC CAAACT
	M13	TG CTG AGT ATG TAG AGA CGC CAAACT
	M14	TG CTG AGT ATG TAG AGA TGT CAAACT
	M15	TG CTG AGC ATG TAC AGA TGC CAAACT
	M16	CTG AGT ATG TAG AGA TGC
	17 nt	TG AGT ATG TAG AGA TGC CAAACT
	18 nt	CTG AGT ATG TAG AGA TGC CAAACT
	19nt	G CTG AGT ATG TAG AGA TGC CAAACT
	20nt	TG CTG AGT ATG TAG AGA TGC CAAACT

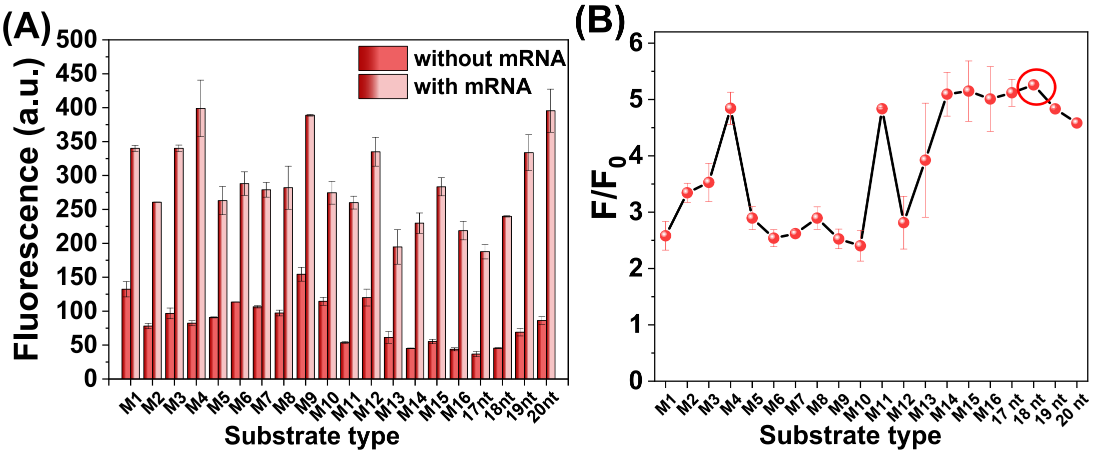

Figure S5. (A) Fluorescence intensity and (B) signal-to-noise ratio (F/F_0) given by different substrate strands. F and F_0 are the fluorescence intensities at 520 nm in the presence and absence of *Survivin* mRNA, respectively. [*Survivin* mRNA] = 20 nM; [Substrate] = 100 nM; [MnO_2 nanosheets] = 2.5 μg/mL; [GSH] = 0.5 mM; [Cas12a] = 50 nM; [crRNA] = 100 nM; [Reporter] = 100 nM. The optimized substrate strand is the one with 18-nt length, which is defined as Substrate in Table S1.

6.2. Substrate concentration

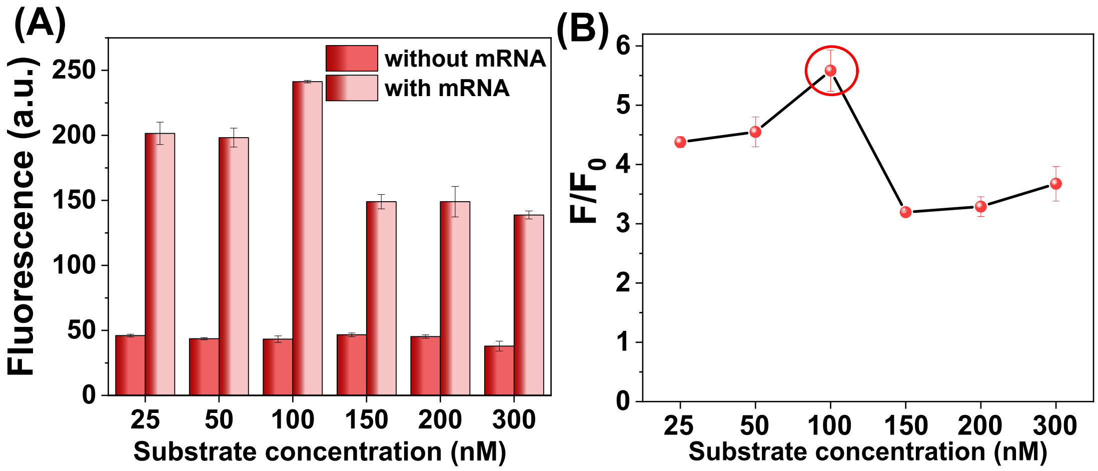

Figure S6. (A) Fluorescence intensity and (B) signal-to-noise ratio (F/F_0) given by the sensing systems containing different concentrations of Substrate. [*Survivin* mRNA] = 20 nM; [MnO_2 nanosheets] = 2.5 μg/mL; [GSH] = 0.5 mM; [Cas12a] = 50 nM; [crRNA] = 100 nM; [Reporter] = 100 nM. The optimized Substrate concentration is 100 nM.

6.3. Amount of MnO_2 nanosheets

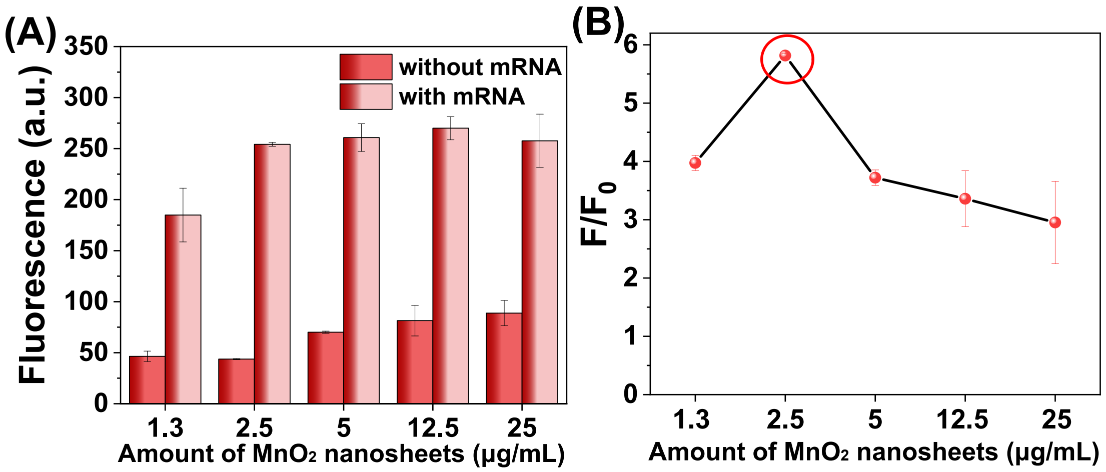

Figure S7. (A) Fluorescence intensity and (B) signal-to-noise ratio (F/F_0) given by the sensing systems containing different amounts of MnO_2 nanosheets. [*Survivin* mRNA] = 20 nM; [GSH] = 0.5 mM; [Substrate] = 100 nM; [Cas12a] = 50 nM; [crRNA] = 100 nM; [Reporter] = 100 nM. The optimized concentration of MnO_2 nanosheets is 2.5 μg/mL.

6.4. crRNA concentration

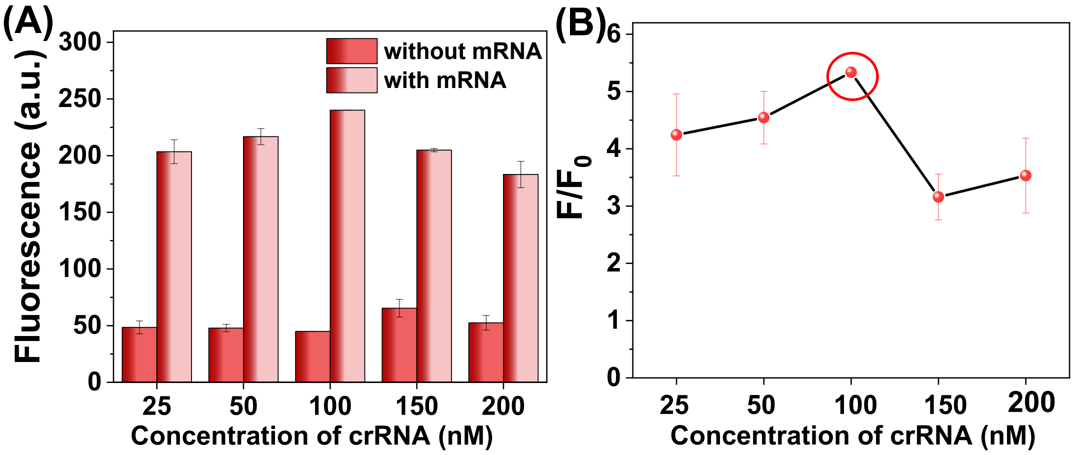

Figure S8. Variance of (A) fluorescence intensity and (B) signal-to-noise ratio (F/F_0) with crRNA concentration. [*Survivin* mRNA] = 20 nM; [MnO_2 nanosheets] = 2.5 µg/mL; [GSH] = 0.5 mM; [Substrate] = 100 nM; [Cas12a] = 50 nM; [Reporter] = 100 nM. The optimized crRNA concentration is 100 nM.

6.5. Cas12a concentration

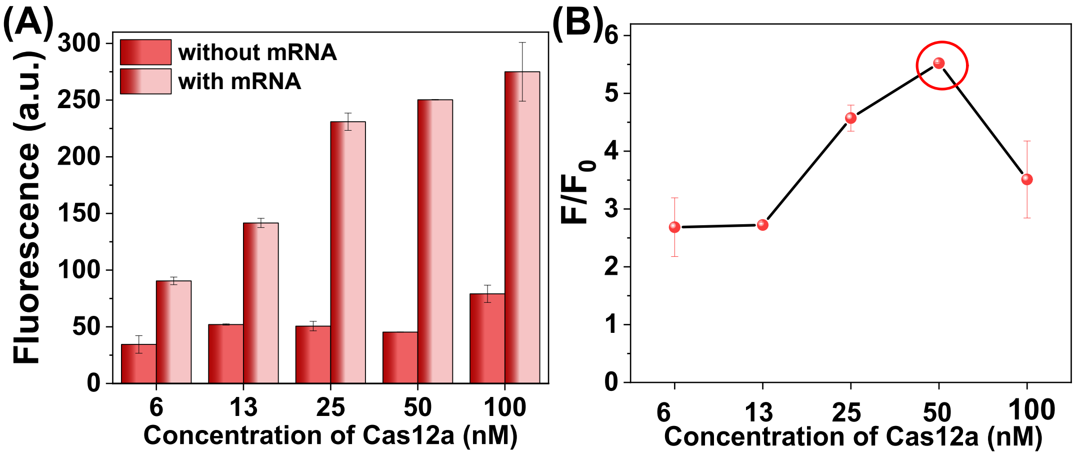

Figure S9. Variance of (A) fluorescence intensity and (B) signal-to-noise ratio (F/F_0) with Cas12a concentration. [*Survivin* mRNA] = 20 nM; [MnO_2 nanosheets] = 2.5 µg/mL; [GSH] = 0.5 mM; [Substrate] = 100 nM; [crRNA] = 100 nM; [Reporter] = 100 nM. The optimized Cas12a concentration is 50 nM.

6.6. Reporter concentration

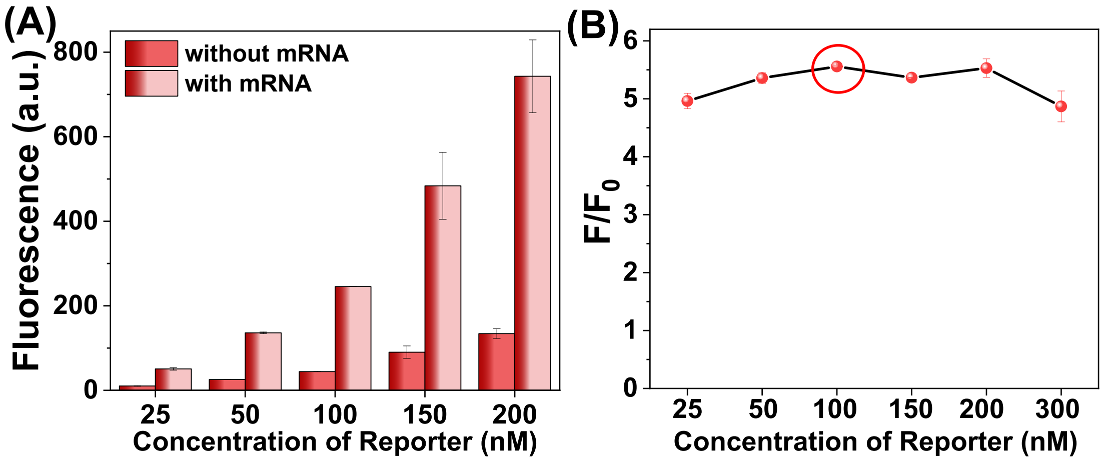

Figure S10. Variance of (A) fluorescence intensity and (B) signal-to-noise ratio (F/F_0) with Reporter concentration. [*Survivin* mRNA] = 20 nM; [MnO_2 nanosheets] = 2.5 μg/mL; [GSH] = 0.5 mM; [Substrate] = 100 nM; [Cas12a] = 50 nM; [crRNA] = 100 nM. The optimized Reporter concentration is 100 nM.

6.7. Reaction time

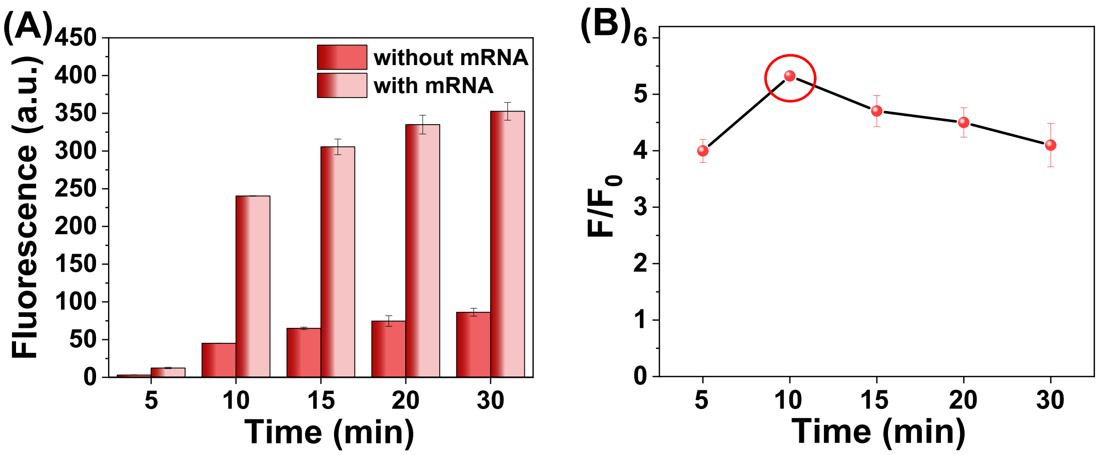

Figure S11. Variance of (A) fluorescence intensity and (B) signal-to-noise ratio (F/F_0) with reaction time. [*Survivin* mRNA] = 20 nM; [Substrate] = 100 nM; [MnO_2 nanosheets] = 2.5 μg/mL; [GSH] = 0.5 mM; [Cas12a] = 50 nM; [crRNA] = 100 nM; [Reporter] = 100 nM. The optimized reaction time is 10 min.

7. Comparison of our *Survivin* mRNA detection method with other reported ones

Table S3. Comparison of several *survivin* mRNA detection methods

Method	Operation and procedures	Detection limit	Linear range	Reference
Fluorescence	Highly integrated, biostable, and self-powered DNA motor	1.2 nM	2.5 nM - 20 nM	1
Fluorescence	Photo-gated and self-powered three-dimensional DNA motors	25.6 pM	30 pM - 50 nM	2
Fluorescence	mRNA-activated multifunctional DNAzyme nanotweezer	1.5 nM	4.5 nM - 25 nM	3
Fluorescence	Accelerated DNA cascade HCR reaction	10.9 pM	50 pM - 50 nM	4
Fluorescence	MnO_2/Cas12a nanoprobe-based fluorescenct sensor	67 pM	200 pM - 50 nM	This work

8. Cytotoxicity evaluation of MnO_2@Cas12a nanoprobe

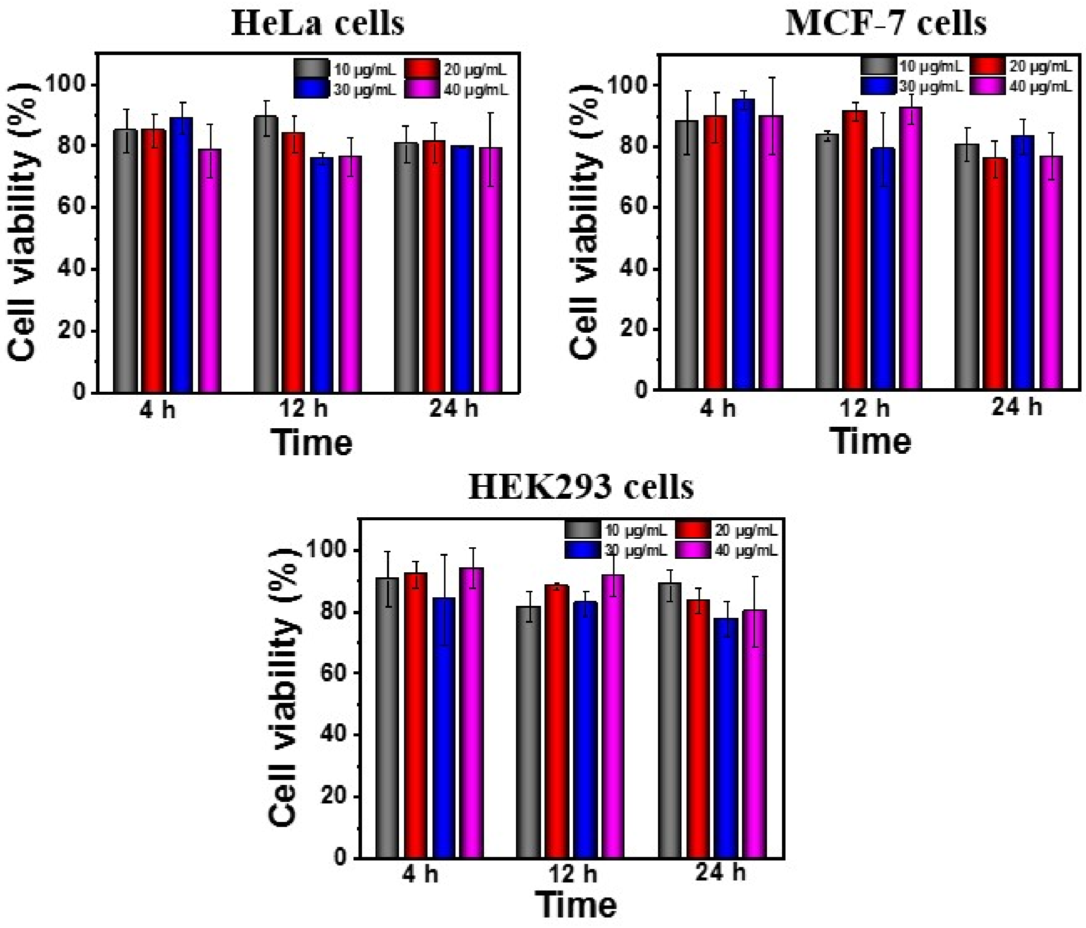

Figure S12. Viabilities of HeLa, MCF-7 and HEK293 cells treated with different concentrations of MnO_2@Cas12a nanoprobe (10 μg/mL, 20 μg/mL, 30 μg/mL and 40 μg/mL of MnO_2 nanosheets) for different time.

9. Feasibility of MnO_2@Cas12a for intracellular mRNA-imaging

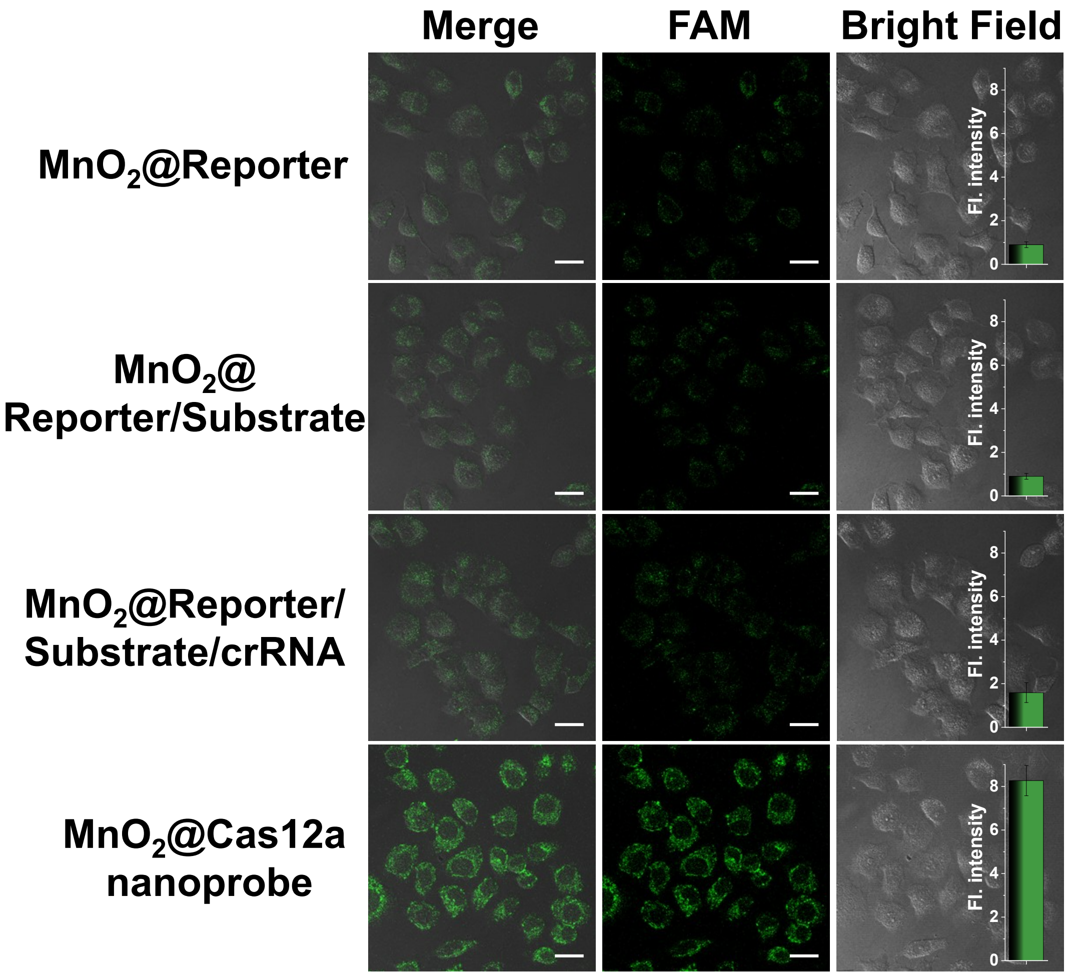

Figure S13. CLSM images of HeLa cells incubated with different agents for 2 h. Scale bar: 25 μm; magnification: 60 ×. Data represent means ± SD (89, 94, 92, 92 cells were measured from top to bottom).

10. Optimization of incubation time for cell imaging

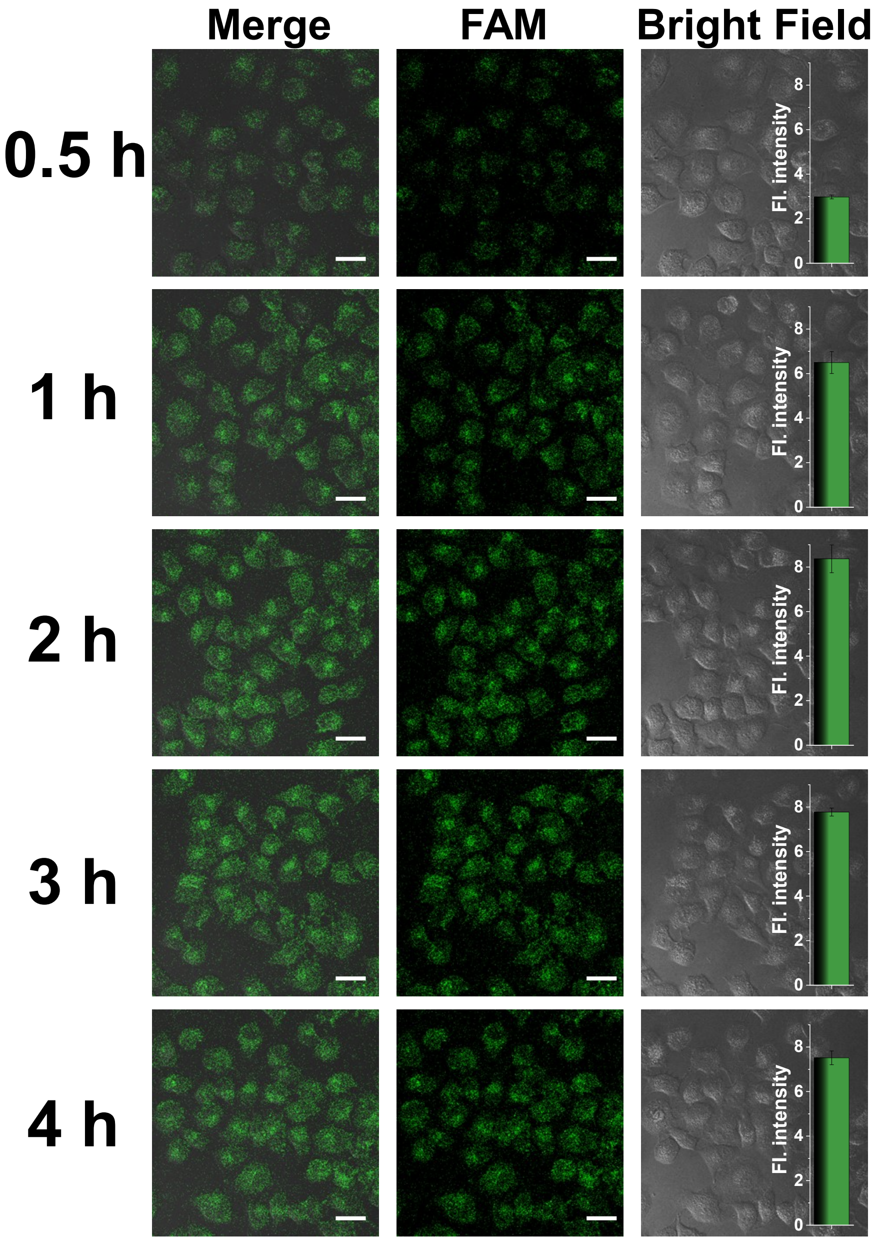

Figure S14. CLSM images of HeLa cells after incubation with MnO_2@Cas12a nanoprobe for different time. Scale bar: 25 μm; magnification: 60 ×. Data represent means ± SD (90, 90, 94, 96, 92 cells were measured from top to bottom).

References

(1) Wang, J.; Wang, D. X.; Tang, A. N.; Kong, D. M. Highly integrated, biostable, and self-powered DNA motor enabling autonomous operation in living bodies. *Anal. Chem.* **2019**, *91*, 5244-5251.

(2) Liu, Y. H.; Gao, J. L.; Liu, J. X.; Liu, D.; Fang, W. K.; Zheng, B.; Tang, H. W.; Li, C. Y. Photo-gated and self-powered three-dimensional DNA motors with boosted biostability for exceptionally precise and efficient tracing of intracellular survivin mRNA. *Biosens. Bioelectron.* **2021**, *190*, 113445.

(3) He, M.; He, M.; Nie, C.; Yi, J.; Zhang, J.; Chen, T.; Chu, X. mRNA-activated multifunctional DNAzyme nanotweezer for intracellular mRNA sensing and gene therapy. *ACS Appl. Mater. Interfaces* **2021**, *13*, 8015-8025.

(4) Ren, K.; Xu, Y.; Liu, Y.; Yang, M.; Ju, H. A responsive "nano string light" for highly efficient mRNA imaging in living cells via accelerated DNA cascade reaction. *ACS Nano* **2018**, *12*, 263-271.
